# DeltaBreed: A BrAPI-centric breeding data information system

**DOI:** 10.1371/journal.pone.0324104

**Published:** 2025-12-12

**Authors:** Shawn C. Yarnes, Nick Palladino, Dave J. Meidlinger, David R. Philips, Heather M. Sweeney, Shahana A. Mustafa, Matthew L. Mandych, Sam Bouabane, Tim E. Parsons, Tyler J. Slonecki, Dongyan Zhao, Trevor W. Rife, Bryan J. Ellerbrock, Chaney Courtney, Peter Selby, Lukas A. Mueller, Mirella Flores-Gonzalez, Johan S. Aparicio, Khaled Al-Sham’aa, Sebastian Raubach, Jean-Luc Jannink, Edward S. Buckler, Craig T. Beil, Moira J. Sheehan

**Affiliations:** 1 Breeding Insight, Cornell Institute for Biotechnology, Cornell University, Ithaca, New York, United States of America; 2 Plant and Environmental Sciences Department, Clemson University, Florence, South Carolina, United States of America; 3 School of Integrative Plant Science, Cornell University, Ithaca, New York, United States of America; 4 Boyce Thompson Institute, Cornell University, Ithaca, New York, United States of America; 5 Department of Plant and Agroecosystem Sciences, University of Wisconsin–Madison, Madison, Wisconsin, United States of America; 6 Genetic Innovation Program, International Center for Agricultural Research in the Dry Areas (ICARDA), Cairo, Egypt; 7 Information & Computational Sciences Department, The James Hutton Institute, Dundee, Scotland, United Kingdom; 8 Plant, Soil and Nutrition Laboratory, Robert W. Holley Center for Agriculture & Health, United States of America Department of Agriculture-Agricultural Research Service, Ithaca, New York, United States of America; Amity University, INDIA

## Abstract

DeltaBreed is a unified breeding data management system designed by Breeding Insight (BI, Cornell University) to serve the wide diversity of USDA-ARS specialty crop and livestock breeding programs. DeltaBreed has a RESTful microservice architecture that utilizes the BrAPI v2.1 Java Test Server as its primary database. The system is interoperable with many BrAPI-compliant applications (BrApps), including Field Book v6.1.0, and is continually aligned with the most recent BrAPI specifications (BrAPI v2.1). Here we describe the features of DeltaBreed v1.0, a minimum viable product, and how we aligned data capture and validation with community standards. We highlight the modules for management of germplasm, observation variables, experiments and observations, genotypic sample submission, and a prototype genomic database that supports polyploid and multiallelic genomic data, as well as SNP data. Several test cases are illustrated to demonstrate the successes and challenges of interoperability with other open-source BrAPI-enabled software packages. We also discuss expansion and enhancement plans for future DeltaBreed versions, as well as outline possible solutions to known limitations. To our knowledge, DeltaBreed is the first species-agnostic, fully BrAPI-compliant breeding data management system built for transactional use.

## Introduction

**Fig 1:**
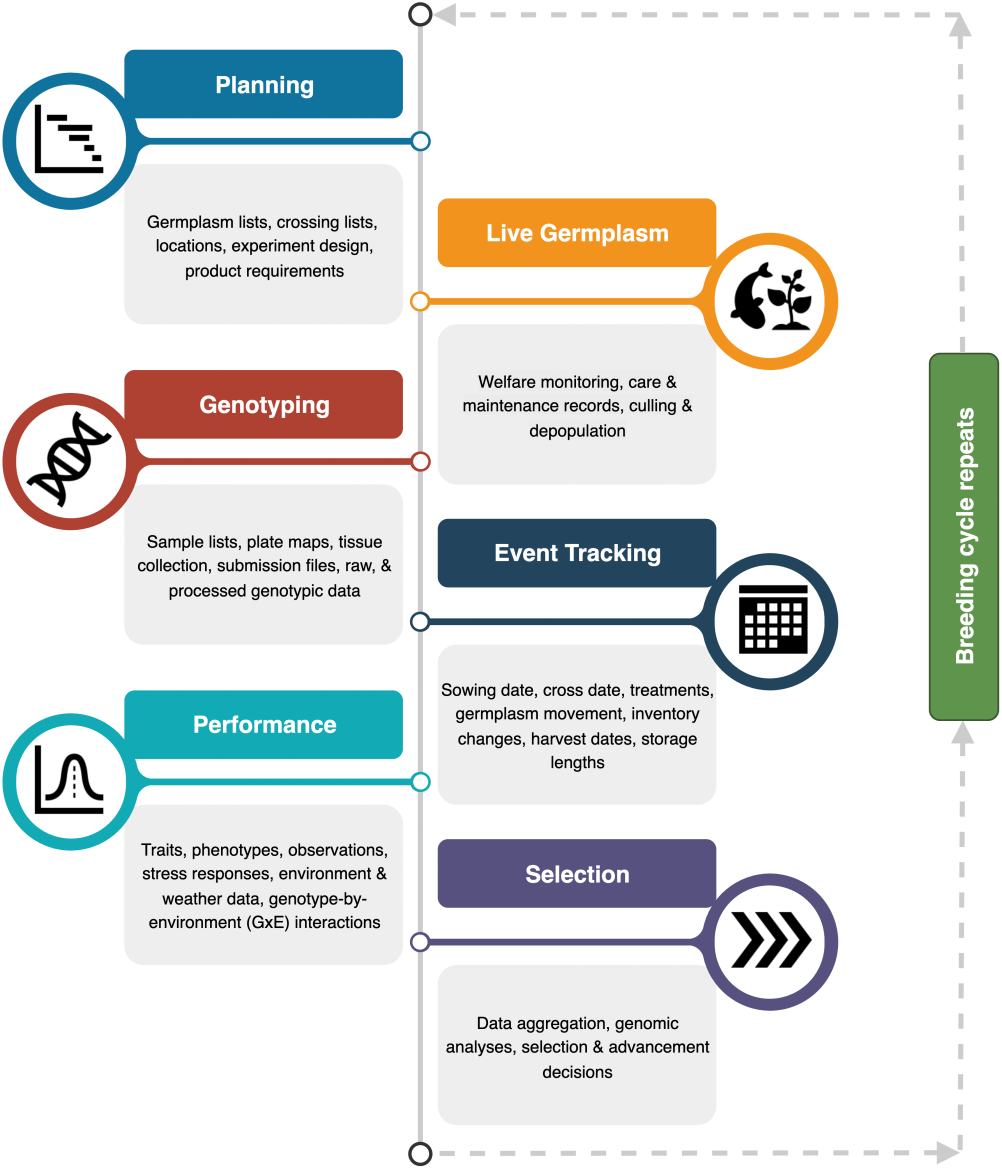
Common activities in breeding programs within a single breeding cycle. Breeding cycles generate and maintain various data types to facilitate data-driven decisions for genetic improvement. At each activity (colored boxes) are different objectives within the breeding cycle: Planning, Live Germplasm, Genotyping, Events, Performance, and Selection. Data type examples are provided for each of the six activities (grey boxes below each activity).

Plant and animal breeding programs require the creation, management, and integration of a multitude of data types to effect genetic gains in elite accessions of crops and livestock. Breeding cycles, regardless of the species or cycle length share a set of common activities that generate or aggregate data. These data need to be effectively stored and easily recalled by breeders to guide their breeding decisions (Fig 1).

Before each breeding cycle, breeding records are reviewed, analyzed, and aggregated to plan for planting and rearing in the season ahead. The data reviewed includes pedigrees, traits of interest, prior crosses made, and the location and amount of material in storage for available germplasm. Also included in planning are the objectives (i.e., product requirements) necessary to achieve genetic gain, optimal trial design for achieving statistical power, and considerations regarding the experimental environments. Collecting detailed records of weather data and/or controlled environment data across all locations in an experiment can help breeders detect genotype-by-environment interactions that will impact selection. The primary goal of most breeding cycles is to generate new genetic variation through planned crosses. Having accurate lists of germplasm available for crosses, along with a crossing plan developed before the cycle begins, helps breeders target their crossing activities to a narrow window of fertility. Some programs plan crosses using pedigree-based Estimated Breeding Values (EBVs), while others use performance and/or genetic data. Genetic information is extremely valuable for planning crosses by allowing the breeder to predict phenotypes and modulating allele frequencies in populations, and to monitor the movement of QTL and other genomic regions from one germplasm pool to another. Genotyping involves creating sample collection lists, plate maps, submission files, receiving raw data or processed data from service providers, and performing quality control (QC).

Once germplasm is alive in fields, greenhouses, tanks, or pens, the effort shifts to management as the germplasm matures and continues until the germplasm is no longer living. Breeders and their staff collect data to document management events like chemical applications (pesticides and herbicides), mitigating treatments (fertigation and antibiotics), and tracking when germplasm is moved from one location to another (common for livestock and honeybees). Dates of management activities like sowing, crossing, births, genotype sampling, harvest, depopulation/culling are also recorded. Germplasm is often evaluated for performance based on multiple phenotypic traits. Observation data requires clear descriptions of how to collect trait data, support for raw data recording, and documented relationships to relevant data, like genotypic samples. Post-harvest performance data are essential for evaluating nutritional quality, as well as determining the optimal storage length and conditions for raw food products.

Altogether, these activities and data types allow breeders to track and monitor progress, respond and mitigate issues, prepare staff and equipment, collect observations and genetic information, and bring data types together to make data-driven and performance-informed analyses and decisions. That is, if they can find and retrieve all data types when needed for quick decisions. Data management tools are essential. In large, industrial breeding programs for maize or soybeans, companies purchase or license sophisticated data management platforms, such as PRISM, Genovix, and Blomeo (and others), often at a substantial annual fee of tens of thousands of US dollars. These platforms support activities across the entire breeding pipeline (from pre-breeding to commercialization) to manage data in a unified platform. At large breeding companies, scientists may only be familiar with a subset of the database system’s data types and activities. In stark contrast, public plant and animal breeders must perform all duties alone or with a small number of supporting staff. Furthermore, public breeding is vastly underfunded and relies on scientists from the US Department of Agriculture – Agricultural Research Service (USDA-ARS) and US land-grant universities to obtain grants or receive royalties from the sale of released varieties for funding. With a typical annual operating budget of $200,000 to $300,000, expensive software licenses are often out of reach [1], leaving many to use software licensed by their employer at no additional cost to them (e.g., Microsoft Excel) as a data management solution. Most breeders utilize Excel in some capacity, as it is convenient for organizing and sharing data with others. Excel has some data validation capabilities, but many breeders do not utilize them or do not know how to use them, such that data stored in Excel often contains errors that a formalized database would have flagged for cleanup or disallowed completely. In summary, there is a need for an open-source, user-friendly, and modular database system that supports any plant or animal breeding program, regardless of team size or budget.

DeltaBreed (RRID:SCR_026678) is a unified information system built by Breeding Insight (BI, RRID:SCR_026645) for managing findable, accessible, interoperable, and reusable (FAIR) breeding data [2,3]. The DeltaBreed GitHub repository contains the technical details of the system, including how to deploy DeltaBreed. BI developed DeltaBreed to support the wide diversity of USDA-ARS specialty plant and animal breeding programs. To date, few USDA-ARS specialty breeding programs utilize a system other than Excel to manage various data types, including germplasm, pedigrees, observation variables, experiments, observations, genotype samples, and genotyping results. USDA-ARS specialty breeding programs require considerable logistical efforts by small research teams managing trialing, advancing, and selecting improved germplasm. Funding, staffing, and time constraints [4] place these programs at a disadvantage in terms of data management and selection power. Breeding data are characterized by disparate data types and increasingly large data volumes that are difficult to store, query, join, and analyze without support and infrastructure for server-based solutions. BI strives to fill this gap by developing and administering the DeltaBreed v1.0 web application for USDA-ARS breeders and public partners.

DeltaBreed v1.0 is a minimum viable product (MVP): the most basic version of software with just enough features to be usable by early adopters to validate the core product ideas. In this case, DeltaBreed v1.0 is the most streamlined species-agnostic system required to manage breeding data originating from BI’s collaborating breeding programs and germplasm repositories [5]. Breeders have shared substantial phenotypic and pedigree data with BI to assist in the validation of genotyping panels [6–9], support marker-trait associations, and perform population structure analysis [10–12]. We and others [13] have found that data from trialing experiments are often ambiguous due to undefined variables, murky pedigrees, and missing identifiers. DeltaBreed v1.0 was built as a foundation to ensure that breeders moving forward can intuitively maintain FAIR breeding data that supports compatibility and interoperability with apps developed by BI and others, as well as improve public access to data.

DeltaBreed is an open-source application (Apache License, v2) that uses the Breeding Application Programming Interface (BrAPI v2.1), a community-defined standard [14], as the primary way to transmit breeding data between different software applications. The BrAPI v2.1 technical specification provides standardized API definitions for DeltaBreed to successfully communicate data between a suite of BrApps (BrAPI-compliant Applications). The release of DeltaBreed v1.0 demonstrates a BrAPI-centric approach that leverages open-source software from unrelated or loosely related initiatives, is adaptable to new BrAPI endpoints, and is fully backward compatible with prior BrAPI versions. While not yet fully realized, seamless interoperability between BrApps is now an attainable goal.

## Results

### System architecture

**Fig 2:**
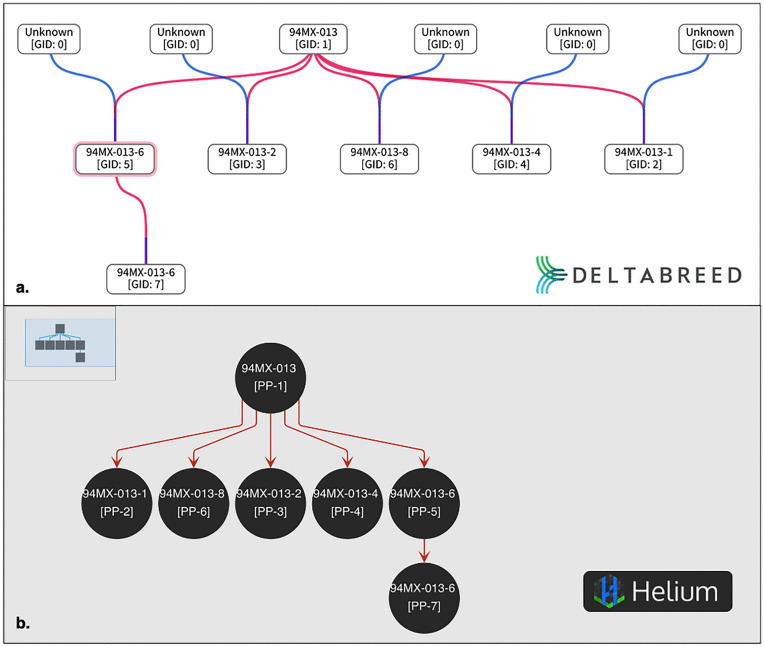
DeltaBreed v1.0 modular software architecture. Users can interact with DeltaBreed v1.0 data through the web interface and via external BrApps. REST APIs, primarily BrAPI, are used to communicate breeding data among various web services, including DeltaBreed-associated databases. DeltaBreed v1.0 communicates with two BrAPI databases and uses a custom database to handle data not supported by BrAPI, like user account management. Disparate data types are handled by connected subsystems with optimized data architectures suitable to the data storage needs. BrAPI logo republished under a CC BY license, with permission from Peter Selby, original copywrite 2016.

DeltaBreed v1.0 has a RESTful (Representational State Transfer) microservice architecture (Fig 2), which differentiates it from other open-source breeding information systems that employ single-tier application frameworks [15–18]. As illustrated in Fig 2, BI has taken a modular approach to developing DeltaBreed v1.0, using REST APIs (primarily BrAPI), to communicate data between web services, building the system around a single database. The advantage of modularity is that services can easily be added, updated, or swapped as needed and with minimal impact on users. However, optimizing performance is a known challenge of the RESTful microservice approach. The latency between systems and the architecture of individual services impact overall performance, which can be exacerbated by poor internet connectivity. DeltaBreed v1.0 utilizes a Redis cache, an in-memory data store that serves as a caching layer in front of databases, to enable fast data access and user interface (UI) load and wait times. Without an efficient method for cashing data, long lists or high-density data could experience significant lag times, frustrating users.

#### Interoperability test case 1: BrAPI Java Test Server & BreedBase.

The DeltaBreed v1.0 system requires a fully BrAPI-compatible database for storing breeding data, specifically BrAPI-core, BrAPI-germplasm, and BrAPI-phenotyping data specifications. Breeding Insight has successfully integrated with two such databases, BreedBase [16,19] and the BrAPI Java Test Server (BJTS). BreedBase is a standalone system with a single-tier architecture (2.3M lines of code) that uses BrAPI mainly for communication with external apps. The BJTS is a fully deployed example of the BrAPI specification, designed for the BrAPI community to test the implementation of BrAPI endpoints [20]. As the BJTS was previously untested, BI initially made use of BreedBase to store DeltaBreed production data. While DeltaBreed v1.0 and BreedBase are interoperable via BrAPI, we achieve shorter load times when DeltaBreed v1.0 is connected to the lighter-weight BJTS (0.5M lines of code). DeltaBreed v1.0 now utilizes the BJTS as its primary database. This test demonstrates that a microservice architecture supported by BrAPI permits changes to the database schema that would be more difficult in a single-tier application.

#### User experience (UX).

**Fig 3:**
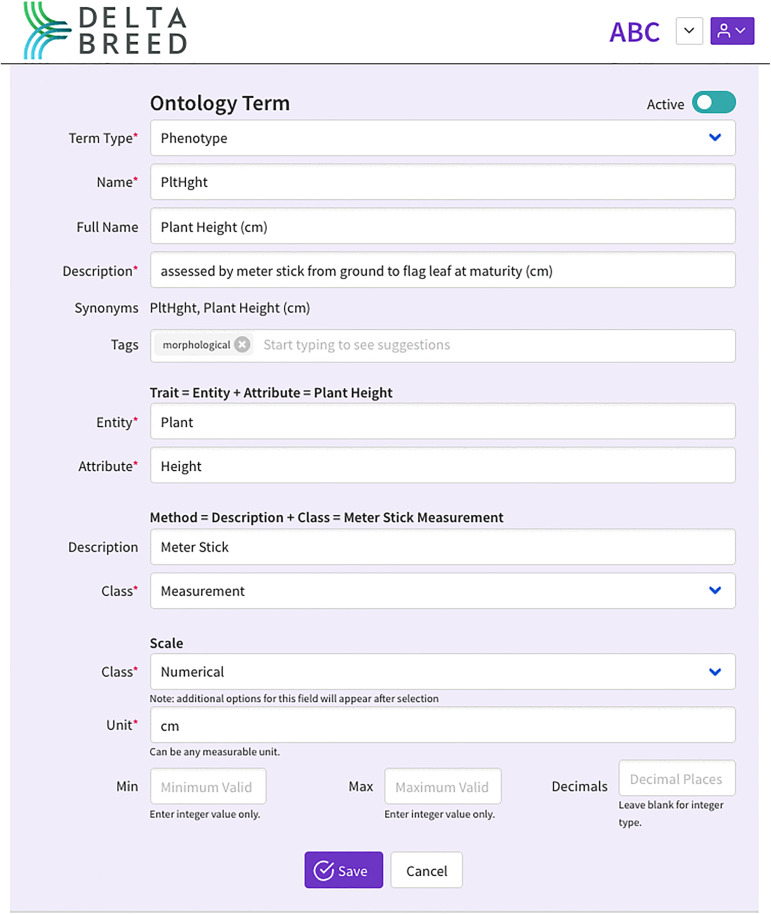
A screenshot of DeltaBreed v1.0 experiment and observation file import module displaying data validation and error messaging. DeltaBreed v1.0 has clear and actionable UI error messaging, as demonstrated in the experiment import module. Error messages appear as a banner across the top of the windowpane. In this example, there are several data errors detected in the Experiment and Observation import file. An error table indicates to users to the exact row(s) in the import file that needs correction.

Despite having modular architecture, DeltaBreed v1.0 provides a unified UI/UX (user interface/user experience) for managing diverse data types. Users can learn DeltaBreed 1.0 features without being aware of BrAPI or the underlying services. The initial users of DeltaBreed v1.0 are BI coordinators and data curators who support USDA-ARS partner programs to adopt FAIR data management practices. BI coordinators have been instrumental in ensuring that DeltaBreed v1.0 is easy to use and well-documented by providing comprehensive UX testing and assisting with the creation of the user manual and training materials. DeltaBreed v1.0 establishes data standards and validations previously absent in USDA-ARS partner programs. Common to all DeltaBreed v1.0 data management modules are simple workflows, defined data requirements, informative error messaging, transparent data views, and cohesive formatting of template and download files. An example screenshot from DeltaBreed v1.0 displaying an error message as a banner is shown in Fig 3.

#### Authorization and program privacy.

DeltaBreed v1.0 helps the USDA meet its open data requirements by balancing privacy, security, and openness. DeltaBreed v1.0 is a private system administered by BI. Invited users authenticate themselves using their ORCID (Open Researcher and Contributor ID), a digital identifier that integrates with DeltaBreed v1.0 through the open authorization (OAuth 2.0) protocol. OAuth 2.0 is an open standard protocol for software account authorization that enables third-party applications to access resources (like user data) on behalf of a user without needing to share the user’s credentials (like passwords). In this case, ORCID’s authorization server issues an authorization code or an access token to DeltaBreed, which allows the user access to only their protected data in DeltaBreed. The implementation of ORCID’s OAuth 2.0 allows users to grant limited access to their DeltaBreed data while keeping their core credentials private from DeltaBreed. Users interact with breeding data from within their associated program(s). Different program roles and permissions allow for various levels of data openness within a program, including program administration, read-only, and experiment collaboration. Data openness is entirely controlled by breeding program administrators and can be employed at various levels to suit the lead breeder’s needs.

### Ontology

**Fig 4:**
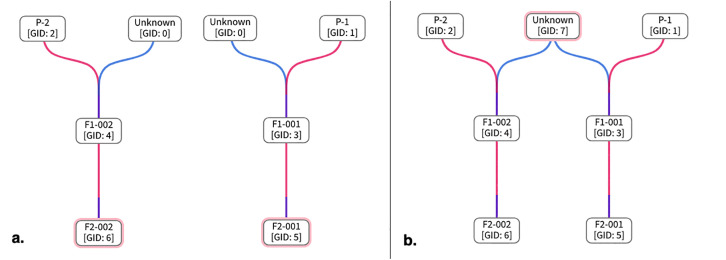
DeltaBreed v1.0 ontology module screenshot of a Plant Height observation variable.

All breeding programs use a collection of variables to describe agronomic, morphological, physiological, quality, and stress observations. Variable ambiguity is a significant barrier to digital data capture, data reuse, and the aggregation of phenotypic data across projects, locations, and time. Over the past decade, a global community effort has been underway to formalize observation variables into controlled vocabularies, known as crop ontologies. The DeltaBreed v1.0 ontology module allows users to create custom observation variables that establish standards for observation validations. DeltaBreed v1.0 defines observation variables by adhering to the crop ontology conceptual module [21,22] and the BrAPI specification [14]. The DeltaBreed v1.0 ontology, specifically the scale class, establishes standards and validations for observations. For example, observations defined by numerical variables are validated to exclude alphabetic characters, while observations for text variables are not validated at all (S1 Table). When data validations fail, an error message banner appears with detailed language on the error and what the user should do to fix it. For batch uploading of trait ontologies, users can download an Excel import template within DeltaBreed v1.0. The template includes a readme tab to guide trait curation. A screenshot of DeltaBreed v1.0 UI for single-trait management of a trait “Plant Height” is shown in Fig 4. “Plant Height (cm)”is the full name of a phenotypic observation variable, and the variable name, in this case “PltHght, is a short identifier, limited to 16 characters to minimize space in tabular and graphical views. The variable is described with a detailed methodology and tagged as “morphological” to group with related variables. The ontology includes the crop ontology conceptual model, which defines variables by their trait (plant height), method (meter stick measurement), and scale (numerical (cm)).

DeltaBreed v1.0 ontology module screenshot of a Plant Height observation variable (see S1 File for data). The “Term” type is selected as ‘Phenotype’. The “Name” is a 16-character-limited abbreviation set by the breeder and minimizes display space in tabular and graphical views. “Full Name” is the full human-readable trait name set by the breeder. The “Description” field indicates how the trait should be measured with as much detail as to convey the procedure completely. The module also allows for the display of “Synonyms”, in this case, it shows the “Name” and the “Full Name” fields but could include other synonyms such as ‘PH’ or ‘PlHt’. The “Tags” field is a way of grouping or categorizing traits for improved human searching and can be anything the breeder chooses for such groupings. For example, ‘post-harvest’ could be a tag used by breeders for crop samples undergoing laboratory testing. The ontology includes the crop ontology conceptual model, which defines variables by their trait (plant height), method (meter stick measurement), and scale (numerical (cm)). A “Trait” is defined by concatenating the observed organism’s part (the “Entity”) and what “Attribute” is being described or measured. In this example, the “Entity” is the entire ‘plant’ and the “Attribute” being measured is ‘height’. The “Method” is defined by concatenating how the attribute is measured (called “Description”) and the method type or “Class”. In this example, a ‘Meter Stick’ is being used as a ‘Measurement’ method “Class”. Other method “Class” options include ‘Estimation’, ‘Observation’, ‘Counting’, and ‘Computation’, which may have other required settings in the “Scale” portion. Additional information is required for a “Measurement” method to indicate that the scale “Class” is ‘Numerical’ and the “Unit” of measurement is ‘cm’. Other “Scale” classes include ‘Date’, ‘Duration’, ‘Nominal’, ‘Ordinal’, and ‘Text’ that vary in their data validation requirements (see S1 Table and S7 File). Red asterisks indicate fields that cannot be left blank.

### Germplasm management

The DeltaBreed v1.0 concept of germplasm is very broad by design. Germplasm is defined as any biological unit (gamete pool, individual, clone, or population) capable of reproduction via any breeding method (DeltaBreed offers >60 pre-defined methods with the option to add customized ones). Drawing from the ICIS Genealogy Management System model [23], each DeltaBreed v1.0 germplasm record is linked by breeding method to its progenitors through unique IDs. DeltaBreed v1.0 assigns both UUIDs (Universally Unique IDs) and human-readable sequential numbers called GIDs (germplasm IDs) to every germplasm record. The dependence on unique UUIDs alleviates the need to adhere to complex naming logic to convey germplasm details, including pedigree connections, and allows for duplicate named entities that are, in fact, separate records (a common occurrence in clonally propagated species). Names make poor unique identifiers because they often break down due to common spelling mistakes, differing capitalization, and the inconsistent use of spaces in names. There is also a tendency for names to evolve over time, such as during the selection process and at variety release or when acquiring germplasm from collaborators. Names are also unsuitable for uniquely differentiating clonal germplasm, which commonly inherits the name of its progenitor. For example, grape varietal clones are generally uniquely named only after the discovery of phenotypically important somaclonal variation (e.g., Pinot Gris). To support breeding for any species, DeltaBreed v1.0 accepts all germplasm naming conventions, permits name duplicates, and allows for custom breeding methods. Users can download an Excel import template in DeltaBreed v1.0 for loading germplasm records (S8 File). The template includes a readme tab to guide users in preparing their data for successful importation.

#### Interoperability test case 2: UI integration of BrAPI pedigree viewer.

**Fig 5:**
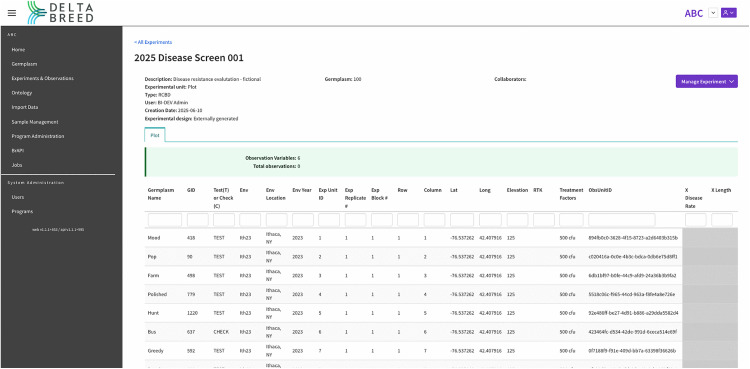
Screenshots of DeltaBreed v1.0 pedigree viewer module when there is an unknown parent in germplasm records.

Genealogical records are universal to all breeding programs, and breeders want to visualize relatedness in pedigree trees. We have fully integrated an interactive pedigree viewer into DeltaBreed v1.0 by adapting a previously existing open-source BrAPI Pedigree Viewer developed by the Boyce Thompson Institute [24]. In this case, we found that integrating this BrApp was faster than developing a comparable feature *de novo*. Most of BI’s development effort was focused on refining and enhancing the visualization in the DeltaBreed v1.0 UI. We added the GID to the germplasm name and developed an unknown germplasm placeholder, Unknown [GID: 0] (Fig 5A). Parental unknowns, especially the male parent, are relatively common in pedigree records. DeltaBreed v1.0’s business logic differentiates Unknown [GID: 0] from other germplasm records to prevent spurious connections in the pedigree tree and to avoid the misleading impression that a single record is making an outsized genetic contribution (Fig 5B). As demonstrated in Fig 5A, the pedigree with a placeholder Unknown [GID:0] shows the correct pedigree tree of two fictional germplasm that are unrelated, F1-002 and F1-001. DeltaBreed v1.0 has business logic to differentiate Unknown [GID: 0] from other germplasm records to prevent spurious connections in the pedigree tree and to avoid the misleading impression that a single record is making an outsized genetic contribution. When ‘Unknown’ is treated like an ordinary germplasm record with a GID greater than 0, unrelated lines can appear erroneously related (Fig 5B).

An example of two unrelated germplasm entities whose records are missing the identification of a male parent in a biparental cross. (A) The correct and accurate pedigree display of the two unrelated lines is made possible by DeltaBreed business logic by using a true Unknown [GID:0] placeholder (see S2 File for data). (B) An incorrect and misleading pedigree display of the same two unrelated lines showing that they share a parent called ‘Unknown GID:7’ (see S3 File for data). When ‘Unknown’ is treated like an ordinary germplasm record with a GID greater than 0, F1-001 and F1-002 appear erroneously as half-siblings.

#### Interoperability test case 3: Helium pedigree viewer BrAPI connection.

**Fig 6:**
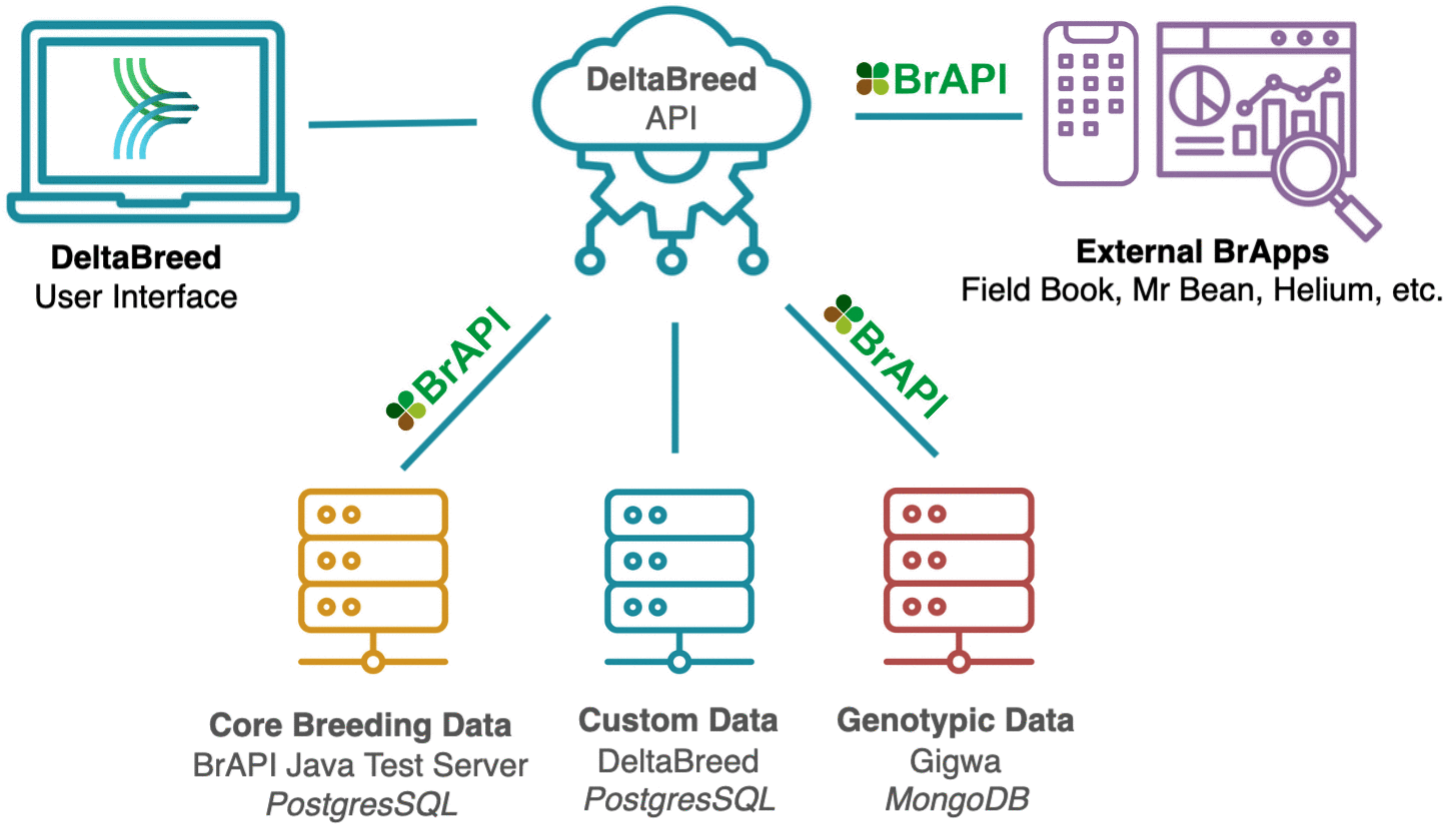
Screenshots of a pedigree view comparing DeltaBreed v1.0 and Helium for the same pecan individual.

Helium is a pedigree viewing BrApp developed by The James Hutton Institute (JHI) [25,26] that includes commercial libraries incompatible with DeltaBreed v1.0’s Apache 2 license. Although license incompatibility limits our ability to integrate Helium fully, we recently collaborated with JHI to allow Helium users to authorize and pull pedigree data directly from a DeltaBreed v1.0 program. DeltaBreed v1.0 users can use Helium filter capabilities, specifically the filter by accession number (DeltaBreed v1.0 GID), to visualize the genealogy of a single germplasm record (Fig 6B). As illustrated in Fig 6, the same example pedigree is shown in the DeltaBreed v1.0 UI and Helium’s UI, demonstrating the interoperability provided by BrAPI.

A fictionalized expanded pedigree of a pecan accession named ‘94MX-013-6’ with GID:5 displayed in the (A) DeltaBreed v1.0 and in the (B) Helium pedigree viewers (see S4 File for data). 94MX-013-6 [GID:5] is one of five open pollinated offspring of 94MX-013 [GID:1] and has one clonal descendant 94MX-013-6 [GID:7]. Note three key differences between A) DeltaBreed v1.0 and B) Helium visualizations: 1) Helium displays the germplasm identifier as 94MX-013-6 [PP:5], which includes a program code “PP” that DeltaBreed strips from its UI view. 2) DeltaBreed v1.0 highlights the selected germplasm with a light pink outline, while Helium does not. 3) DeltaBreed v1.0 can denote the pollen source for the open pollenated lines with Unknown [GID:0], while Helium does not. Genealogy Courtesy of Warren Chatwin, USDA-ARS Pecan Somerville, TX. Genealogy represented under a CC BY license, with permission from Warren Chatwin, original copyright 2025. Helium logo represented under a CC BY license with permission from Sebastian Raubach, original copyright 2019.

However, limits to interoperability remain. DeltaBreed v1.0 programs with more than a few thousand germplasm records cannot use Helium’s default pedigree retrieval because thousands of pedigree nodes are not meaningfully visualized in a single view, and load times are prohibitive for practical use. Additionally, DeltaBreed v1.0’s business logic for germplasm names causes some confusion when pedigrees are rendered in Helium and other BrApps. DeltaBreed v1.0 displays a simple germplasm name in the UI but musts save germplasm names to the database as a concatenation (i.e., name + program code + GID) to ensure compatibility with BrApps that do not differentiate data by programs and require unique germplasm names. Although derived from DeltaBreed v1.0, the program code and GIDs rendered in the Helium UI (see rigid brackets in Fig 6B) are not details a DeltaBreed v1.0 user would expect or desire to be included in a germplasm name and appear as an artifact of interoperability. Also, note that Helium does not recognize DeltaBreed v1.0’s ‘Unknown [GID: 0]’ (Fig 6A), in part because there is no BrAPI mechanism to transmit the special case of an unknown germplasm record.

### Experiments and observations

DeltaBreed v1.0 allows users to create and append single or multi-environment experiments. Experiments describe the spatiotemporal arrangement of germplasm (GIDs) into observation units (ObsUnitIDs) to make observations and detect statistical differences in phenotypic response. Experiments can also describe non-randomized arrangements, such as nurseries or crossing blocks, that may have purposes beyond phenotyping. The DeltaBreed v1.0 experiment concept aligns closely with Minimal Information About a Plant Phenotyping Experiments (MIAPPE) standards [27], including most terminology and BrAPI mappings (see S2 Table). Users can download an Excel import template within DeltaBreed v1.0 for loading experiments and trials (S9 File). The template includes a README tab to guide users in preparing their data for successful importation.

**Fig 7:**
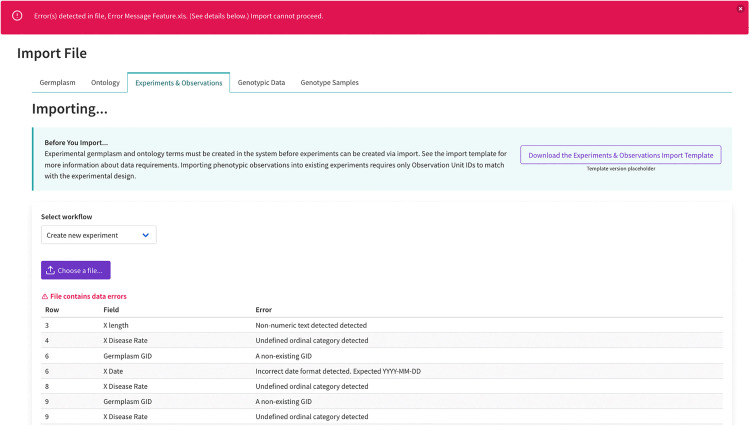
Screenshot of DeltaBreed v1.0 experiments and observations user interface. Displayed is an experiment view in DeltaBreed v1.0 before observations have been made (see S5 File for data). Each table row represents an observation unit, in this case a plot, with its unique ObsUnitID and associated human-readable germplasm record (GID). The grey cells represent pending observations with no recorded values. Experimental observation variables (e.g., “XLength”) are defined in the DeltaBreed v1.0 ontology module.

Active experiment management in DeltaBreed v1.0 is a multi-step process that begins with creating an experiment prior to data collection and ends with appending observational data. The DeltaBreed v1.0 UI screenshot in Fig 7 shows an example experiment loaded with trial metadata, field layout, and traits for which observations are planned. In this example, the observations are greyed out as observation data has not been collected or uploaded. Interoperability test cases 4 and 5 both involve active experiment management using the Field Book v6.1.0 mobile app [28,29]. Field Book v6.1.0 is a widely used BrApp for recording phenotypic observations and measurements on handheld Android devices like phones and tablets. Field Book v6.1.0 is part of the PhenoApps project, and BI has worked closely with the PhenoApps team to improve its BrAPI interoperability.

#### Interoperability test case 4: DeltaBreed v0.8, BreedBase, and Field Book v5.0.

Before integrating the BJTS, DeltaBreed v0.8 used BreedBase for all data storage due to its BrAPI v1.3 compliance. BI integrated several BrApps and Field Book v5.0 with BreedBase to support active 2021 experimental data collection by USDA-ARS blueberry breeders. We used the DeltaBreed v0.8 UI to establish observation variables, which were stored in BreedBase, and utilized the BreedBase UI to establish studies before data collection. We also enabled Field Book v5.0 to pull studies and observation variables from BreedBase and sync observations made in Field Book v5.0 back to BreedBase after data collection. This effort served as a successful proof of concept for multi-application BrAPI integration but highlighted unforeseen limitations in the process of syncing observations back to BreedBase.

Prior to this integration, Field Book files had to be manually moved in and out of the app, which allowed users to view and QC the raw data before committing it to BreedBase. The ability to sync data directly from Field Book v5.0 into BreedBase effectively removed this QC step and revealed limitations in BreedBase BrAPI transaction handling. Firstly, BreedBase did not enforce atomic (i.e., complete transactions), so network errors frequently resulted in partial upload of observation batches with no warning or error messaging. Secondly, when multiple users synced data from Field Book v5.0 back to BreedBase, or a single user repeatedly synced, only the most recently upload observations were available in the UI and study download files. This resulted confusion, because user expectations differed from the system records. Manual curation of flat files was ultimately necessary to resolve missing, duplicated, and erroneous observation values arising from inadvertent partial observation uploads, repeated uploads by single users, and extensive uploads to the database by multiple data.

#### Interoperability test case 5: DeltaBreed v1.0 & Field Book v6.1.0.

**Fig 8:**
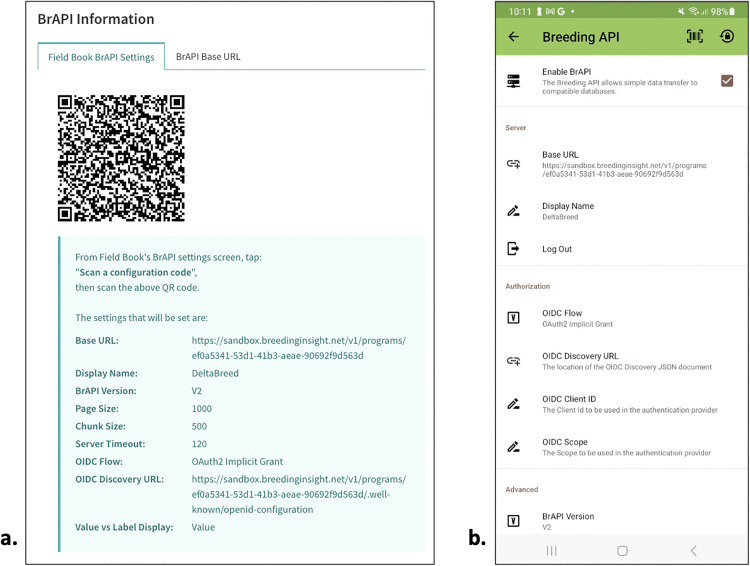
Screenshots of DeltaBreed v1.0 and BrAPI v2.1 interoperability with the Android mobile app, Field Book v6.1.0. Side-by-side screenshots of **(A)** DeltaBreed v1.0 Field Book BrAPI settings and **(B)** Field Book v6.1.0 BrAPI settings after auto-configuration with DeltaBreed. DeltaBreed v1.0 auto-configuration simplifies inter-app connections so that experimental datasets and observation variables can be pulled from DeltaBreed v1.0 into Field Book v6.1.0 for data collection. Field Book screenshot republished under a CC BY license, with permission from Trevor Rife, original copyright 2023.

After DeltaBreed integrated the BJTS, we developed new features to improve DeltaBreed v1.0-Field Book v6.1.0 interoperability. Early versions of DeltaBreed required Field Book v6.1.0 users to type in a long URL to begin authentication into their DeltaBreed breeding program, leading to typos, connection errors, and user frustration. To simplify the steps to connect Field Book v6.1.0 to specific DeltaBreed programs, we developed a configuration setting in Field Book (Fig 8) and created a program QR code in DeltaBreed (Fig 8A). Now users can scan the program QR code with the Android device running Field Book v6.1.0 to auto-configure the BrAPI connection (Fig 8B). This workflow eliminates manual configuration and speeds connection time, especially when multiple tablets are in use. The process of selecting observation variables for data collection in Field Book v6.1.0 was also streamlined. Observation variables associated with DeltaBreed v1.0 experimental datasets are now automatically selected in Field Book v6.1.0, reducing the need to manually choose or search through long lists of variables in the field to find the traits for the day’s data collection effort.

To avoid time-consuming resolution of data discrepancies encountered in the previous test case 4, and to allow users to QC raw data before committing it to the database, DeltaBreed v1.0 does not accept data from external apps via BrAPI. Observations are uploaded by flat file, ensuring that the data collector or breeder has curated the raw observations before appending an experiment with observations. The 2024 field season included USDA-ARS alfalfa and hemp as beta testers with Breeding Insight support. The breeders managed field experiments in DeltaBreed v1.0, pulled environments and observation variables to Field Book v6.1.0 via BrAPI, and recorded their observations in Field Book v6.1.0. When the data collection is complete, they will share their Field Book v6.1.0 files with Breeding Insight so that observations can be loaded into DeltaBreed v1.0. In the future, breeders will be trained to complete the data collection workflow independently. Thus far, both groups have reported successful data collection activities in the field (Zhanyou Xu and Tyler Gordon, personal communications).

#### Interoperability test case 6: MrBean & QBMS.

**Fig 9:**
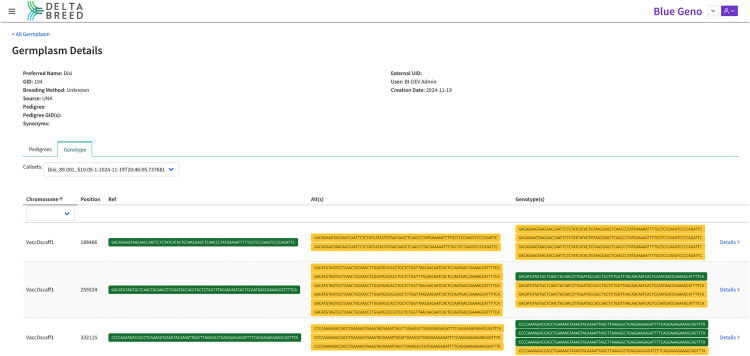
Screenshot of MrBean’s UI for descriptive plots using potato data pulled from DeltaBreed v1.0 via QBMS and BrAPI. The center-left panel displays a scatterplot of all germplasm evaluated for total yield in the Vignette1 potato dataset [34]. The center-right panel displays a boxplot of total yield across five environments (the location “Hancock” across years 2015−2019). Notice in the scatter plot that the selected germplasmName is appended with program code and GID, [TATER-273]. Similarly, studyName in the boxplot is appended with program code and experiment ID. These appends are hidden from users on the DeltaBreed v1.0 UI and are artifacts of DeltaBreed v1.0 interoperability logic, also described with Helium integration (see Fig 4). See S6 File for data. MrBean logo republished under a CC BY license, with permission from Johan Aparicio, original copyright 2023.

MrBean is an R-Shiny app designed for breeders with no R programming experience to visualize and model spatial trends in single and multi-environment experiments [30,31]. MrBean works with a core set of R packages, including the QBMS package [32,33], which allows MrBean to access BrAPI endpoints in compliant databases. In collaboration with MrBean and QMBS developers, BI has updated the MrBean and the QBMS code bases to enable users to authenticate to DeltaBreed v1.0 and pull experiment and observation data via BrAPI. For this text case, we loaded the Vignette1 [34] dataset for potato into DeltaBreed v1.0. Next, we used QBMS to pull the dataset from DeltaBreed and display it in MrBean. As illustrated in the screenshot shown in Fig 9, we successfully used MrBean to generate descriptive statistics and plots and perform *lme4* model fitting [35] from Vignette1 dataset in DeltaBreed, without having to download the data, reformat it, and load it directly into MrBean. The ability to bring data from DeltaBreed v1.0 into MrBean allows breeders to make data-informed decisions for culling, advancement, and selection in their breeding program, thereby leveraging the robust R programming under MrBean without needing to be proficient R users. The proven interoperability with MrBean also alleviates the need for DeltaBreed to develop and implement a separate spatial analysis package. While additional effort is needed to integrate other MrBean modeling options with DeltaBreed v1.0, this test case demonstrates its feasibility and value to breeding programs.

### Genotype sample management

The DeltaBreed v1.0 sample management module supports the BI genotyping sample submission process. BI has facilitated the genotyping of more than 71,000 samples for our partner programs through a multi-step process that involves collecting sample submission details, submitting the genotyping order to the vendor, receiving and processing FASTQ files from the vendor, and producing SNP datasets for the breeder. Essential to this process are DeltaBreed-generated UUIDs that are compatible with all systems involved, including human-readable sample names. Samples can be associated with either germplasm records IDs (GIDs) or experimental observation units IDs (ObsUnitIDs), the latter of which is more information-dense. Observation units connect samples to germplasm records, spatiotemporal details, and phenotypic observations. Users can download an Excel import template within DeltaBreed v1.0 for loading sample lists for genotyping (S10 File). The template includes a README tab to guide users in preparing their data for successful importation.

#### Interoperability test case 7: DeltaBreed v1.0 and Gigwa.

**Fig 10:**
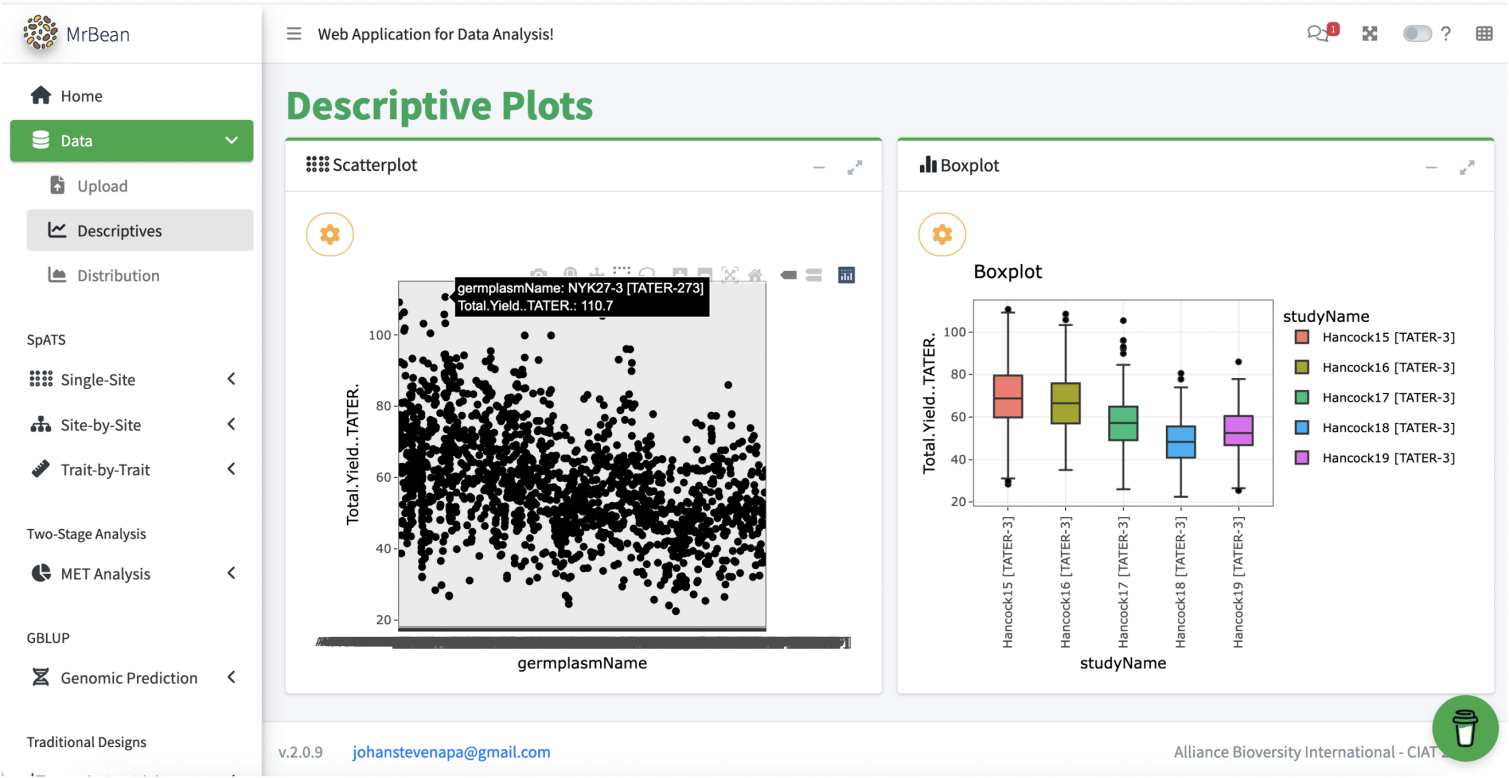
Screenshot of DeltaBreed v1.1 prototype visualization of microhaplotypes in the germplasm record view for a blueberry accession called “Dixi”. The Germplasm Details viewer shows the genotype of an autotetraploid blueberry (2n = 4x = 48) accession called ‘Dixi’ [GID:104]. The accession was genotyped on the 3K DArTag Blueberry panel (Blackberry_DArTag_BI_Cornell_University (1.0), [9]) and microhaplotypes were discovered, curated, and displayed at each targeted locus (only 3 loci are shown in this example). At each marker locus, the “Ref” column shows the microhaplotype allele that matches the reference genome exactly for the target sequence and surrounding sequence, the “Alt(s)” column shows the microhaplotype allele that matches known alternative alleles at the target locus by design, and the “Genotype(s)” displays the four microhaplotypes detected at on each of the four homologous chromosomes at the indicated locus in the genome. The ‘Dixi’ cultivar harbors four copies of “Alt” alleles at locus VaccDscaff1_188466, one copy of the “Ref” and three copies of the “Alt” alleles at locus VaccDscaff1_255524, and three copies of “Ref” and one copy of the “Alt” alleles at locus VaccDscaff1_332115.

BI is actively developing a complete genotyping workflow and has implemented a DeltaBreed v1.1 prototype to integrate the Genotype Investigator for Genome-Wide Analysis (Gigwa, RRID:SCR_017080; https://southgreen.fr/content/gigwa) into the DeltaBreed architecture (see Fig 2) for genotypic data storage [36–38]. Unlike the previous test cases where DeltaBreed v1.0 communicated to external apps, the test case here was to fully integrate Gigwa under the DeltaBreed v1.1 UI, such that a user might never realize that an external app is even connected. The prototype is a proof of concept that demonstrates Gigwa’s support for future DeltaBreed versions to load SNP or short-read sequencing data in Variant Call File (VCF) format, and to enable microhaplotype visualization. A screenshot of the Germplasm display with a proof-of-concept UI for Genotype illustrates how an autotetraploid species, blueberry in this case, would have microhaplotype data displayed in DeltaBreed (Fig 10). While storage of genetic SNP data associated with a breeding program is not novel, the ability to store multi-allelic microhaplotype data is. Microhaplotypes are short sequencing reads of 54–250 bp generated by the DArTag targeted genotyping platform (Diversity Arrays Technology, PLC; [39]). Microhaplotypes comprise a target SNP, as well as off-target ‘discovered’ SNPs, all of which are in complete linkage disequilibrium and thus increase the genetic information acquired at each targeted locus. The increased information content of microhaplotypes is extremely valuable in autopolyploid crop species for calling dosage and assigning genotype phase. Breeding Insight has produced 3K DArTag marker panels for numerous species (https://breedinginsight.org/breeding-solutions/open-source-dartag-marker-panels/, [6,7,9]). Breeding Insight has been collecting and cataloging all discovered microhaplotype alleles for every BI-created panel into a custom database called ‘HaploBase’. As a HaploBase instance is built out for each DArTag panel, future versions of DeltaBreed need to be able to connect the tens of thousands of microhaplotypes back to phenotypes to allow breeders to search for and utilize trait-associated microhaplotypes in their mate selection, germplasm acquisition and maintenance, and variety release activities.

## Discussion

### Overview

DeltaBreed v1.0 demonstrates the power and unique development challenges of open-source community models and standards in the deployment of FAIR breeding data management tools. DeltaBreed v1.0 interoperability test cases confirm that a BrAPI-centric microservice architecture is a nimble approach to incorporate services or swap underlying databases (Test Case 1). Open-source BrApps with compatible licenses can be fully integrated into DeltaBreed v1.0 with less development effort than *de novo* coding (Test Cases 1, 2, 4, 5, 7). While any BrApp is theoretically interoperable with DeltaBreed v1.0, we have found that interoperability with independently developed and maintained BrApps is not guaranteed, and even when the connection is technically possible, the user experience benefits from additional refinement (Test Cases 1–6).

### Performance

BrAPI is integral to DeltaBreed. In addition to facilitating interoperability with independent BrApps, BrAPI facilitates DeltaBreed v1.0 intersystem connections. The BrAPI Java Test Server has proven to be a modestly performant breeding database for DeltaBreed v1.0. The loading time for a single file containing 30,000 aquaculture germplasm records (i.e., individual fish) with extensive pedigree connections took nine hours to load into DeltaBreed v1.0 via the BrAPI Java Test Server, which is an improvement over the 22 hours it took to load the same file into BreedBase. Still, nine hours is not performant enough to accommodate the needs of species already supported in BI’s portfolio, much less accommodate breeding efforts that may include hundreds of thousands of datapoints. The limited performance of the BrAPI Java Test Server has prompted the BI and BrAPI teams to develop a BrAPI Java Production Server (BJPS) with enhanced performance, full CRUD functions (create, read, update, and delete), and atomic transaction handling. Future versions of DeltaBreed will utilize the BJPS and continue to be aligned with the latest version of the BrAPI specifications. New DeltaBreed features, including new delete functions, will be possible with BrAPI v2.2 updates. Breeding Insight is committed to continued efforts with the BrAPI community to expand the flexibility and utility of the BrAPI specification. These and other planned enhancements will enable future DeltaBreed versions to be utilized by major crop and livestock breeders running significantly larger programs or programs that coordinating testing across multiple locations.

### BrAPI interoperability

DeltaBreed v1.0 interoperability is the result of the BrAPI community cross-initiative collaboration. Every DeltaBreed v1.0 interoperability test case described in this manuscript required BI development effort and most prompted ongoing and continuous collaborative efforts with other software initiatives, ranging from bug reporting, to feature requests, code-sharing, and co-working at annual BrAPI Hackathons. We have found that collaborative solutions tend to be most efficient when collaborators benefit by gaining new features and/or new users while remaining focused primarily on their own code bases and development priorities. Collaborative solutions also tend to be generalized to the benefit of the larger breeding community. For example, a DeltaBreed use case may have prompted the implementation of OAuth protocols in Field Book v6.1.0 and Helium, but now that the protocol is in place, it is available to any system.

The BrAPI specification is a community-driven project. We can advocate for changes, like a codified endpoint for unknown parent, but until there is community consensus, this aspect of DeltaBreed business logic is not interoperable via BrAPI. Deciding on endpoints by committee is time-consuming and can take much longer than just creating a custom endpoint that’s needed immediately. In some cases, this is the only way forward as the need is relevant, but the community support is not. During the development of DeltaBreed v1.0, we encountered several other use cases unsupported by the BrAPI v2.0 specification: delete and atomic transactions. Delete endpoints have since been added to BrAPI v2.1 specification, and upcoming DeltaBreed versions will include the delete functions our users require to become independent data managers. Atomic transactions refer to an all-or-nothing batch upload or download event, which prevents partial data transfers that may be interrupted for any number of reasons (see test case 4). Improved transaction handling is an area of ongoing effort with the BrAPI community and will be addressed in the upcoming BJPS.

Additional limitations to interoperability arise from different development cycle times in connectable applications. For example, a BrAPI-compliant app may only be partially BrAPI-compliant (i.e., compliant with prior versions of the BrAPI specification or only specific endpoints) if the developer hasn’t kept up with the BrAPI specification or hasn’t made any changes to the application itself. In these cases, BrAPI v2.1 has refined an endpoint that DeltaBreed is attempting to call, but the connecting system can’t return the data. These cases are easier to correct for interoperability as they tend to require fewer developer hours, and the connections don’t break as easily because the connected app is not under active development. Other software teams move very fast, such as the PhenoApps team that owns Field Book. New versions are released every two to four weeks and they often break interoperability with DeltaBreed. In this case, we find we are constantly fixing DeltaBreed to maintain the connectivity to the latest version of Field Book. Lastly, changes in scope or user needs can be accommodated in our agile project management style, but usually at the expense of some desirable feature that enhances the UI/UX.

Our experience from interoperability tests such as these is that BrAPPs designed to support one use case are rarely seamlessly compatible with BrAPPs designed to support multiple or broader use cases. Helium was not designed with DeltaBreed in mind, and vice versa. Despite the Helium pedigree viewer working well for JHI’s test cases, it was of limited use when connected to a DeltaBreed program with a large volume of germplasm. Testing identified this limitation, and since this manuscript was written, JHI has added the ability to pull a subset of program records based on a single germplasm selection, thereby improving Helium’s interoperability with DeltaBreed v1.0 and other BrAPI-compliant systems.

### Recommendations

Systems, like Deltabreed and others, that warehouse breeding data have complex and largely unavoidable differences in business logic. Differences, particularly in naming rules, pose challenges to the interoperability of community BrApps designed for data collection, analytics, and decision-making. For a BrApp to be interoperable with any system, it needs to be flexible and independent of a system’s logic. Many systems are built on the logic that germplasmName is a globally unique identifier. To better enable DeltaBreed v1.0 interoperability with such systems, we must append name strings returned in BrAPI endpoint responses with additional identifiers. For example, germplasmName is stored in DeltaBreed v1.0 as “name [ProgramCode-GID]”. While this allows DeltaBreed to be interoperable with the business logic of more systems, these name strings are confusing to our native DeltaBreed users in visualizations and analyses performed by external apps. DeltaBreed v1.0 requires multiple BrAPI properties to uniquely identify germplasm (germplasmName, accessionNumber) and experimental datasets (trialName, studyName, observationLevel). As we move forward, we plan to cease using name strings and instead rely on globally unique BrAPI dbIDs for our endpoint responses. We recommend that others building community BrApps do the same. We also recommend that BrApps provide information-rich UIs, including a complete representation of the available BrAPI object properties, especially when providing search or filtering capabilities. More information improves everyone’s user experience and helps support wider interoperability.

### Future enhancements

BI is actively using, maintaining, and improving DeltaBreed in a continuous integration and continuous deployment framework (CI/CD). The system is now available to select USDA-ARS specialty breeding programs. Beta testers have responded positively, especially to interoperability with Field Book v6.1.0. Even when interoperability is not fully UX optimized (Test Cases 4 & 5), our testers enthusiastically adopted Field Book v6.1.0 data collection workflows. Continued improvement of DeltaBreed interoperability and UI/UX with Field Book v6.1.0 and other BrApps is a priority for BI. We are working closely with the PhenoApps team and others to improve and expand processes. However, breeding terminology and human language remain usability sticking points (see S2 Table). Related BrApps may agree on BrAPI communication standards, but they rarely agree on the terminology in their UIs. For example, an “experimental dataset” in DeltaBreed v1.0 is equivalent to a “field” in Field Book v6.1.0 and a “studyName” in MrBean. As BI looks to the future, we are exploring options to make external BrApps appear more seamless with DeltaBreed without interfering with the UI/UX of non-DeltaBreed workflows. In addition to UI/UX improvements, we are developing support channels and step-by-step, workflow-specific training materials for breeders interested in using the system.

### DeltaBreed roll-out and breeder adoption

The release of DeltaBreed v1.0 provides USDA-ARS specialty crop and animal breeders with a species-agnostic, open-source data management system with a friendly UI/UX and low barriers to adoption. The web application makes it easy for users of any operating system to migrate data currently stored solely in Microsoft Excel spreadsheets. Relational databases, such as DeltaBreed, have numerous advantages over spreadsheets. DeltaBreed v1.0 enforces data accuracy through validations, like those based on variable scale class, and maintains data integrity (see S7 File–S10 Files). While spreadsheets can be downloaded from DeltaBreed v1.0 and modified, the data within the system is protected as a source of truth. Breeders of specialty species, particularly perennial, clonal, or livestock species, will find the flexibility they need in DeltaBreed that is not generally found in software built with only annual row crop or major livestock breeders in mind.

## Conclusions

DeltaBreed v1.0 has the potential to accelerate the delivery of improved genetic cultivars to US farmers and producers by providing the data standardization necessary to fully leverage USDA-ARS phenomic and genomic breeding data. DeltaBreed is an open-source relational database system available for organizations to deploy on their preferred IT infrastructure. DeltaBreed’s modular nature and BrAPI-centric design should make the system attractive to universities and small to mid-sized breeding companies that want to standardize their data without reinventing a core platform or committing to long-term financial investment while maximizing customization possible via BrAPI v2.1 interoperability. The flexible architecture, coupled with BrAPI interoperability, offers numerous potential bespoke configurations for customizing breeding workflows and integrating diverse data types into routine breeding decisions in programs.

Planned upgrades to the BJPS will deliver an enterprise system suitable for managing even larger-scale operations, such as the combined breeding effort at USDA-ARS, which spans tens of crops and livestock (>100 crops and ~15 animal) species. The USDA-ARS Office of National Programs invests an average of 1.0 billion US dollars each year to improve crops and food animals through the Crop Production and Protection (CPP) and Animal Production and Protection (APP) budget lines. These funds are used to produce improved germplasm stocks and varieties for public release and free data access to growers, producers, ranchers, nurseries, and farmers [40]. In turn, the 2023 crop returns to U.S. farmers across all commodities for which the USDA-ARS runs a genetic improvement program, generated more than 109 billion US dollars that year alone, a return-on-investment greater than 1:100 per taxpayer dollar spent [41]. Moreover, most USDA-ARS-bred species do not have private-sector genetic improvement enterprises, leaving the USDA-ARS as the primary provider of research and development for specialty crop and livestock improvement in the public sector. The ability to consolidate all breeding management data across the USDA mission areas (which would include the National Arboretum and US Forest Service, both of which engage in breeding efforts) would significantly unify data collection, ensure data integrity and security, and improve cross-collaborations within the agency and for the public for a fraction of the cost that other software-as-a-service systems currently available would charge.

## Supporting information

S1 Table Data validations for observation data available in DeltaBreed v1.0 for variable scale class. (XLSX)

S2 Table DeltaBreed v1.0 terminology definitions and identifiers with related concepts from other breeding community standards. (XLSX)

S1 File Fig 4: Plant height ontology import file. (XLS)

S2 File Fig 5a: Unknown GID 0 import. (XLS)

S3 File Fig 5b: Tangled pedigree import. (XLS)

S4 File Fig 6: DeltaBreed Germplasm with pedigree export. (XLS)

S5 File Fig 7: DeltaBreed experiment export 2025_Disease_Screen_001. (XLS)

S6 File Fig 9: Potato experiment adapted for DeltaBreed. (XLS)

S7 File Supplemental DeltaBreed v1.0 import template files: bi_germplasm_import_template_v11. (XLS)

S8 File bi_experimental_template_v07. (XLS)

S9 File bi_ontology_template_v15. (XLS)

S10 File bi_sample_submission_template_v01. (XLS)
