# Correction: DeltaBreed: A BrAPI-centric breeding data information system

**DOI:** 10.1371/journal.pone.0341691

**Published:** 2026-01-22

**Authors:** Shawn C. Yarnes, Nick Palladino, Dave J. Meidlinger, David R. Philips, Heather M. Sweeney, Shahana A. Mustafa, Matthew L. Mandych, Sam Bouabane, Tim E. Parsons, Tyler J. Slonecki, Dongyan Zhao, Trevor W. Rife, Bryan J. Ellerbrock, Chaney Courtney, Peter Selby, Lukas A. Mueller, Mirella Flores-Gonzalez, Johan S. Aparicio, Khaled Al-Sham’aa, Sebastian Raubach, Jean-Luc Jannink, Edward S. Buckler, Craig T. Beil, Moira J. Sheehan

The images for Figs 2–7 and 9 and 10 are incorrectly switched. The image that appears as Fig 6 should be Fig 2, the image that appears as Fig 4 should be Fig 3, the image that appears as Fig 5 should be Fig 4, the image that appears as Fig 7 should be Fig 5, the image that appears as Fig 2 should be Fig 6, the image that appears as Fig 3 should be Fig 7, the image that appears as Fig 10 should be Fig 9 and the image that appears as Fig 9 should be Fig 10. The figure captions appear in the correct order. The authors have provided a corrected version of figures here.

**Fig 2:**
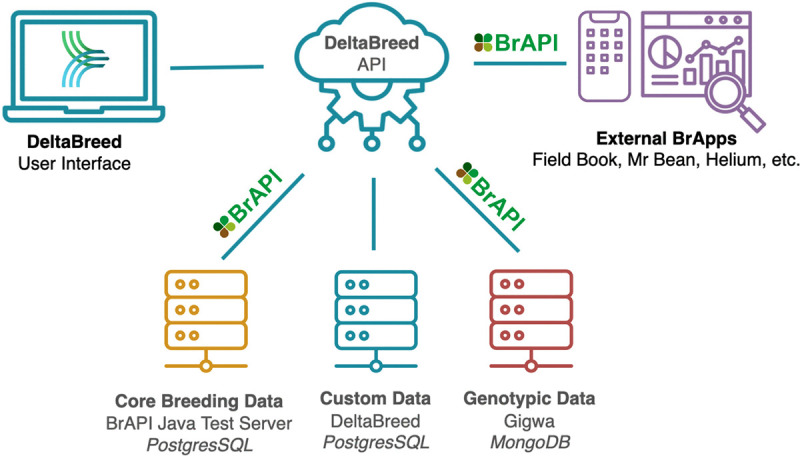
DeltaBreed v1.0 modular software architecture. Users can interact with DeltaBreed v1.0 data through the web interface and via external BrApps. REST APIs, primarily BrAPI, are used to communicate breeding data among various web services, including DeltaBreed-associated databases. DeltaBreed v1.0 communicates with two BrAPI databases and uses a custom database to handle data not supported by BrAPI, like user account management. Disparate data types are handled by connected subsystems with optimized data architectures suitable to the data storage needs. BrAPI logo republished under a CC BY license, with permission from Peter Selby, original copywrite 2016.

**Fig 3:**
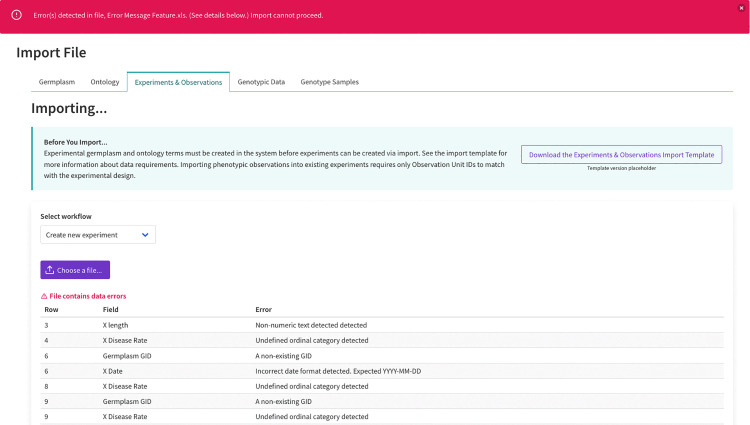
A screenshot of DeltaBreed v1.0 experiment and observation file import module displaying data validation and error messaging. DeltaBreed v1.0 has clear and actionable UI error messaging, as demonstrated in the experiment import module. Error messages appear as a banner across the top of the windowpane. In this example, there are several data errors detected in the Experiment and Observation import file. An error table indicates to users to the exact row(s) in the import file that needs correction.

**Fig 4:**
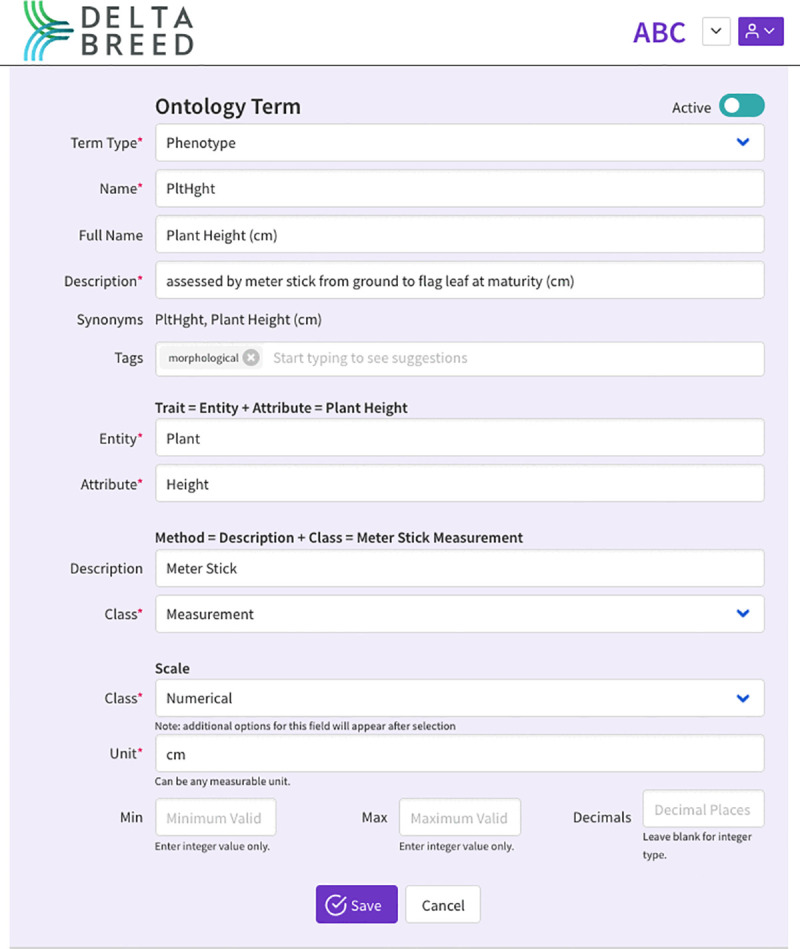
DeltaBreed v1.0 ontology module screenshot of a Plant Height observation variable.

**Fig 5:**
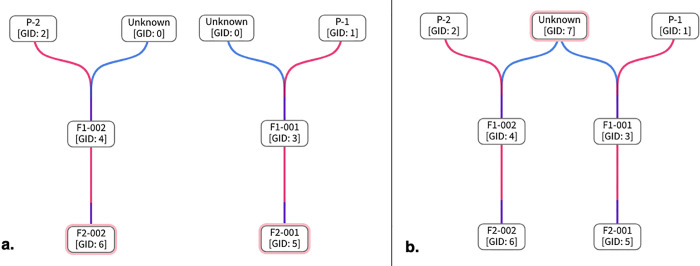
Screenshots of DeltaBreed v1.0 pedigree viewer module when there is an unknown parent in germplasm records.

**Fig 6:**
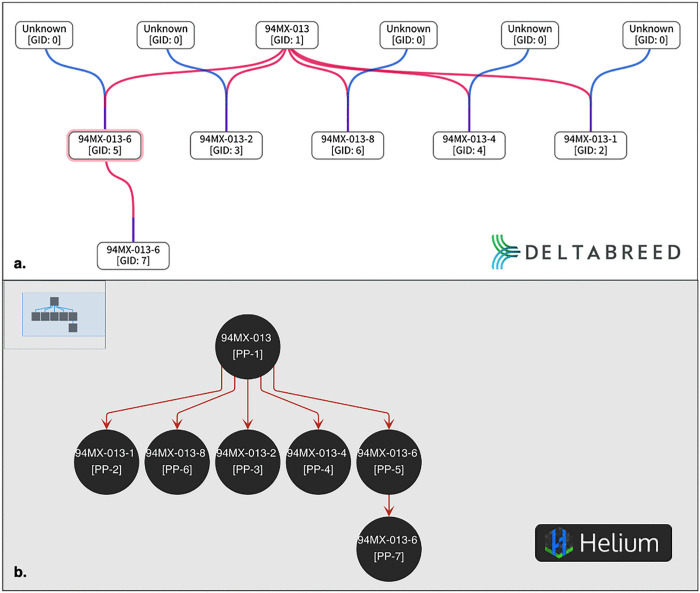
Screenshots of a pedigree view comparing DeltaBreed v1.0 and Helium for the same pecan individual.

**Fig 7:**
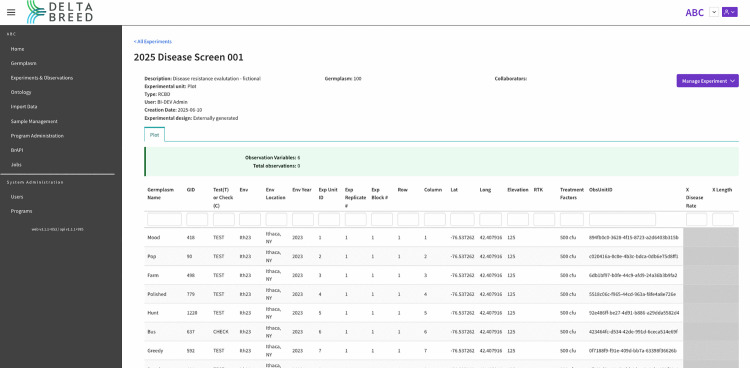
Screenshot of DeltaBreed v1.0 experiments and observations user interface. Displayed is an experiment view in DeltaBreed v1.0 before observations have been made (see S5 File for data). Each table row represents an observation unit, in this case a plot, with its unique ObsUnitID and associated human-readable germplasm record (GID). The grey cells represent pending observations with no recorded values. Experimental observation variables (e.g., “XLength”) are defined in the DeltaBreed v1.0 ontology module.

**Fig 9:**
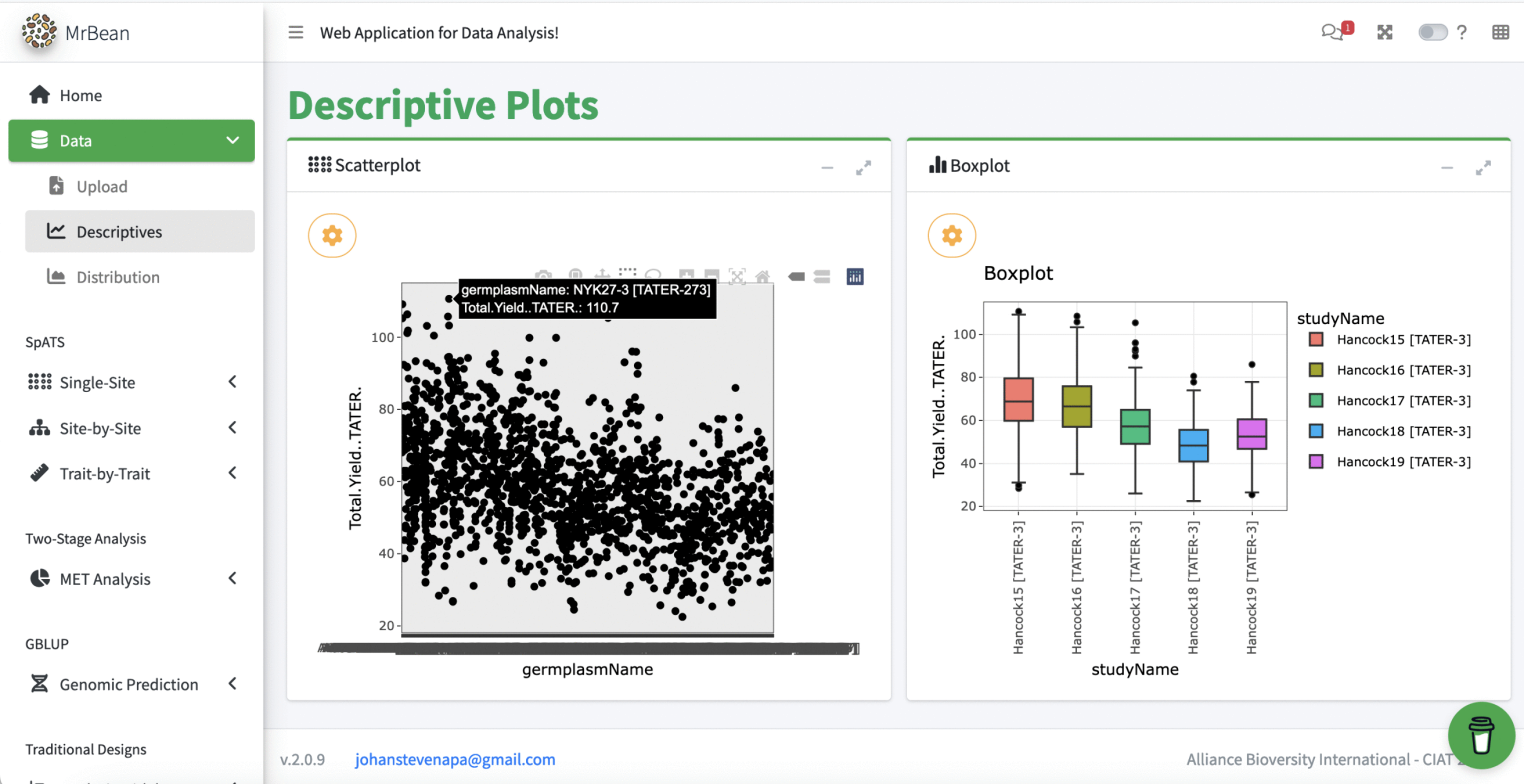
Screenshot of MrBean’s UI for descriptive plots using potato data pulled from DeltaBreed v1.0 via QBMS and BrAPI. The center-left panel displays a scatterplot of all germplasm evaluated for total yield in the Vignette1 potato dataset [34]. The center-right panel displays a boxplot of total yield across five environments (the location “Hancock” across years 2015 − 2019). Notice in the scatter plot that the selected germplasmName is appended with program code and GID, [TATER-273]. Similarly, studyName in the boxplot is appended with program code and experiment ID. These appends are hidden from users on the DeltaBreed v1.0 UI and are artifacts of DeltaBreed v1.0 interoperability logic, also described with Helium integration (see Fig 4). See S6 File for data. MrBean logo republished under a CC BY license, with permission from Johan Aparicio, original copyright 2023.

**Fig 10:**
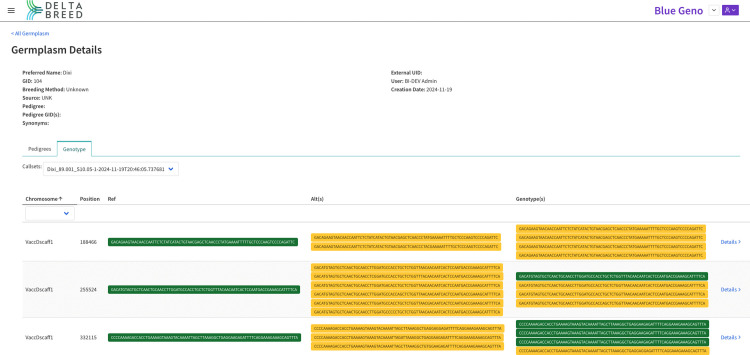
Screenshot of DeltaBreed v1.1 prototype visualization of microhaplotypes in the germplasm record view for a blueberry accession called “Dixi”. The Germplasm Details viewer shows the genotype of an autotetraploid blueberry (2n = 4x = 48) accession called ‘Dixi’ [GID:104]. The accession was genotyped on the 3K DArTag Blueberry panel (Blackberry_DArTag_BI_Cornell_University (1.0), [9]) and microhaplotypes were discovered, curated, and displayed at each targeted locus (only 3 loci are shown in this example). At each marker locus, the “Ref” column shows the microhaplotype allele that matches the reference genome exactly for the target sequence and surrounding sequence, the “Alt(s)” column shows the microhaplotype allele that matches known alternative alleles at the target locus by design, and the “Genotype(s)” displays the four microhaplotypes detected at on each of the four homologous chromosomes at the indicated locus in the genome. The ‘Dixi’ cultivar harbors four copies of “Alt” alleles at locus VaccDscaff1_188466, one copy of the “Ref” and three copies of the “Alt” alleles at locus VaccDscaff1_255524, and three copies of “Ref” and one copy of the “Alt” alleles at locus VaccDscaff1_332115.
